# Branched‐chain α‐keto acids and glutamate/glutamine: Biomarkers of insulin resistance in childhood obesity

**DOI:** 10.1002/edm2.388

**Published:** 2022-11-22

**Authors:** Pinar Gumus Balikcioglu, Catherine Jachthuber Trub, Metin Balikcioglu, Olga Ilkayeva, Phillip J. White, Michael Muehlbauer, James R. Bain, Sarah Armstrong, Michael Freemark

**Affiliations:** ^1^ Division of Pediatric Endocrinology and Diabetes Duke University Medical Center Durham North Carolina USA; ^2^ Duke Molecular Physiology Institute and Sarah W. Stedman Nutrition and Metabolism Center Duke University Medical Center Durham North Carolina USA; ^3^ Division of General Pediatrics University of North Carolina Chapel Hill North Carolina USA; ^4^ Advanced Analytics Division SAS Institute Inc Cary North Carolina USA; ^5^ Division of Endocrinology, Metabolism, and Nutrition Duke University Medical Center Durham North Carolina USA; ^6^ Division of General Pediatrics and Adolescent Health Duke University Medical Center Durham North Carolina USA; ^7^ Department of Family Medicine and Community Health Duke University Medical Center Durham North Carolina USA; ^8^ Department of Population Health Sciences Duke University Medical Center Durham North Carolina USA; ^9^ Duke Clinical Research Institute Duke University Medical Center Durham North Carolina USA

**Keywords:** branched‐chain amino acid, branched‐chain α‐keto acid, childhood obesity, insulin resistance, metabolomics

## Abstract

**Objectives:**

Insulin resistance (IR) in adolescents with obesity is associated with a sex‐dependent metabolic ‘signature’ comprising the branched‐chain amino acids (BCAAs), glutamate/glutamine, C3/C5 acylcarnitines and uric acid. Here, we compared the levels of branched‐chain α‐keto acids (BCKAs) and glutamate/glutamine, which are the byproducts of BCAA catabolism and uric acid among adolescents with obesity prior to and following a 6‐month lifestyle‐intervention program.

**Methods:**

Fasting plasma samples from 33 adolescents with obesity (16 males, 17 females, aged 12–18 year) were analysed by flow‐injection tandem MS and LC–MS/MS. Multiple linear regression models were used to correlate changes in BCKAs, glutamate/glutamine and uric acid with changes in weight and insulin sensitivity as assessed by HOMA‐IR, adiponectin and the ratio of triglyceride (TG) to HDL. In predictive models, BCKAs, glutamate/glutamine and uric acid at baseline were used as explanatory variables.

**Results:**

Baseline BCKAs, glutamate/glutamine and uric acid were higher in males than females despite comparable BMI‐metrics. Following lifestyle‐intervention, α‐keto‐β‐methylvalerate (α‐KMV, a metabolic by product of isoleucine) decreased in males but not in females. The ratio of BCKA/BCAA trended lower in males. In the cohort as a whole, BCKAs correlated positively with the ratio of TG to HDL at baseline and HOMA‐IR at 6‐month‐follow‐up. Glutamate/glutamine was positively associated with HOMA‐IR at baseline and 6‐month‐follow‐up. A reduction in BCKAs was associated with an increase in adiponectin, and those with higher BCKAs at baseline had higher adiponectin levels at 6‐month‐follow‐up. Interestingly those adolescents with higher uric acid levels at baseline had greater reduction in weight.

**Conclusions:**

BCKAs and glutamate/glutamine may serve as biomarkers of IR in adolescents with obesity, and uric acid might serve as a predictor of weight loss in response to lifestyle‐intervention. Differential regulation of BCAA catabolism in adolescent males and females implicates critical roles for sex steroids in metabolic homeostasis.

## Graphical abstract

Insulin resistance (IR) in adolescents with obesity is associated with a sex‐dependent metabolic “signature” comprising the branched‐chain amino acids (BCAAs), glutamate/glutamine, C3/C5 acylcarnitines, and uric acid. Here, we compared the levels of branched‐chain α‐keto acids (BCKAs) and glutamate/glutamine, which are byproducts of BCAA catabolism, as well as uric acid among adolescents with obesity prior to and following a 6‐month lifestyle‐intervention program. We showed BCKAs and glutamate/glutamine may serve as biomarkers of IR in adolescents with obesity, and uric acid might serve as a predictor of weight loss in response to lifestyle‐intervention.

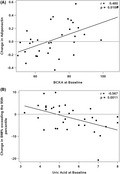

## 1 INTRODUCTION

In previous investigations,^1^ we demonstrated that insulin resistance (IR) in adolescents with obesity associates with a sex‐dependent metabolic ‘signature’ comprising the branched‐chain amino acids (BCAAs), glutamate/glutamine, C3/C5 acylcarnitines and uric acid. The ‘glutamate/glutamine’ analyte represents glutamate, plus an indeterminate contribution from hydrolysis of glutamine during acidic preparation of extracted metabolites for our flow injection‐tandem mass spectrometric assay.^1^, ^2^, ^3^ Weight reduction and increases in insulin sensitivity in response to lifestyle intervention are accompanied by reductions in BCAA, C3/C5 acylcarnitines and glutamate/glutamine and increases in urea cycle intermediates, providing indirect evidence for an increase in BCAA catabolism.^4^ It is currently unclear if an increase in BCAA catabolism is a cause or a consequence (or both) of the heightened insulin sensitivity associated with weight loss in adolescents.

BCAA catabolism is initiated by branched‐chain aminotransferase (BCAT), which facilitates a reversible transamination reaction generating branched‐chain α‐keto acids (BCKAs) including α‐keto‐isocaproate (α‐KIC, from leucine), α‐keto‐β‐methylvalerate (α‐KMV, from isoleucine) and α‐keto‐isovalerate (α‐KIV, from valine).^5^ Recent work in rat and mouse models of obesity and IR demonstrate that pharmacologic activation of BCKA oxidation by the branched chain α‐keto acid dehydrogenase (BCKDH) complex improves insulin sensitivity while lowering circulating BCAAs and BCKAs.^5^, ^6^ Studies in human adults and rats suggest that elevated plasma BCKAs are associated with IR and are potentially better biomarkers for type 2 diabetes (T2D) than BCAAs.^6^, ^7^, ^8^ Furthermore, in a large population‐based cohort from the TwinsUK study, the isoleucine catabolite α‐keto‐β‐methylvalerate (α‐KMV) was found to be the strongest predictive biomarker for impaired fasting glucose.^7^ To the best of our knowledge, no studies have examined the relationship between BCKAs and insulin sensitivity in adolescents with obesity. Thus, it is not known if increases in insulin sensitivity during lifestyle intervention are accompanied by reductions in BCKAs as well as BCAAs, or if baseline levels of BCKAs predict the metabolic response to lifestyle change.

Glutamate is a byproduct of the first step of BCAA catabolism. Studies in adults show that increased fasting plasma levels of glutamate are associated with higher fasting and 2‐h plasma glucose levels during a 75 g‐oral glucose tolerance test^9^ and an increase in the prevalence of T2D.^10^, ^11^ Elevated fasting glutamate levels were also associated with obesity, IR, hypertension and dyslipidaemia.^12^ Furthermore, elevated plasma glutamate levels were associated with increased risks of cardiovascular disease^13^ and subclinical atherosclerosis in adults, even after adjustment for age, sex, body fat mass and visceral fat mass.^14^

Like glutamate, uric acid is a potential risk factor for hypertension and diabetes in adolescents as well as adults,^15^, ^16^, ^17^ but its relationship to BCAA and glutamate metabolism is poorly understood. Hyperuricemia in obesity and insulin resistance is thought to result from decreased insulin‐dependent renal tubular uric acid excretion and/or increased fructose‐dependent uric acid production.^18^, ^19^ However, studies in subjects with gout suggest that increases in glutamate resulting from decreased activity of glutamate dehydrogenase may play a role in the pathogenesis of hyperuricemia.^20^ Thus, elevations in uric acid in adolescents with obesity and insulin resistance might be biologically related to elevations in glutamate.

In this study, we measured BCKAs, glutamate/glutamine and uric acid and explored their relationships individually with changes in weight and insulin sensitivity before and after a lifestyle intervention in adolescents with obesity. *We hypothesized* that (1) BCKAs, glutamate/glutamine and uric acid are associated with insulin resistance at baseline and 6‐month‐follow‐up; (2) weight reduction and improved insulin sensitivity during lifestyle intervention are associated with decreases in BCKAs, glutamate/glutamine and uric acid; (3) baseline BCKAs, glutamate/glutamine and uric acid predict subsequent changes in weight and insulin sensitivity and (4) BCKAs, glutamate/glutamine and uric acid levels are regulated in a sex‐dependent manner. To test these hypotheses, we measured the relevant analytes and applied multiple linear regression models to assess the correlations among BCKAs, glutamate/glutamine and uric acid and weight and surrogate measures of insulin sensitivity as assessed by homeostasis model assessment index of insulin resistance (HOMA‐IR), adiponectin and the ratio of triglyceride (TG) to HDL. In predictive models, the BCKAs, glutamate/glutamine and uric acid at baseline were used as explanatory variables.

## 2 RESEARCH DESIGN AND METHODS

### 2.1 Patient cohort

Participants were identified prior to enrolment in Duke Children's Healthy Lifestyles Program (HLP) and followed prospectively for 6 months. The Duke Children's HLP provides comprehensive clinical care for children and adolescents with overweight and obesity and represents the current standard of clinical care for paediatric obesity treatment (please see Ref.^1^, ^4^, ^21^ for detailed description). Inclusion criteria stipulated that the participant was new to the HLP, ≥12 to 18 years of age and overweight or obese (defined as BMI ≥ 85th percentile for sex and age according to CDC growth charts), and that the participant and at least one parent/guardian were able to speak/read English fluently enough to understand and complete questionnaires and intake forms. Participants were excluded if they had a diagnosis of type 2 diabetes and/or had taken weight‐reducing agents, systemic corticosteroids, atypical antipsychotics, oral contraceptives or medroxyprogesterone within the past 6 months. Participation was terminated if the subject did not provide fasting blood samples within 2 weeks of his/her first clinic visit. Informed consent was obtained from at least one parent/guardian for each participant <18‐year‐old and from participants ≥18‐year‐old. The Duke University Institutional Review Board (IRB) approved the research protocol. Thirty three participants (16 males and 17 females) completed the study and provided fasting plasma samples at baseline and 6 months.^4^

### 2.2 Blood samples

Blood samples were obtained after an 8‐ to 12‐h overnight fast at baseline and 6‐month follow‐up. Plasma was stored at −80°C until analysed.

### 2.3 Anthropometric measurements

Body weight and height were measured by standard methods. Blood pressure was measured twice; the average was used in statistical analyses. Age, sex and height‐specific normal values for children are available at https://www.nhlbi.nih.gov/files/docs/bp_child_pocket.pdf. Body fat percentage was estimated using a Tanita BC‐148 segmental body composition analyser. BMI, BMI percentile (BMI%), BMI z‐score and the percent BMI exceeding the 95th %ile were calculated using the SAS program (https://www.cdc.gov/nccdphp/dnpao/growthcharts/resources/sas.htm). Our cohort consisted of subjects with very high BMI values, making BMI percentile and BMI z score unreliable estimates of the degree of overweight and the response to intervention.^22^, ^23^ Thus, we used ‘BMI percent exceeding the 95^th^ percentile’ for age and sex to track the weight changes over time. The CDC recommends a new classification system recognizing BMI ≥95th percentile as class I obesity, BMI ≥120% of the 95th percentile as class II obesity and BMI ≥140% of the 95th percentile as class III obesity. Class II and III obesity are strongly associated with greater cardiovascular and metabolic risk.^22^, ^23^

### 2.4 Hormone analysis

Hormones were measured using a Meso Scale Discovery Quick Plex electro chemiluminescent imager with assay kits from Meso Scale Discovery (Rockville, MD) including insulin (range 69–50,000 pg/ml) and total adiponectin (range 0.064–1000 ng/ml and samples diluted 1:961). Duplicate measurements had coefficients of variations <10%.

### 2.5 Surrogate measures of insulin sensitivity

As in our previous studies,^1^, ^4^ we used HOMA‐IR, adiponectin and the TG/HDL ratio as surrogate measures of IR. These surrogate measures reflect distinct, but overlapping, components of insulin sensitivity regulated at the level of the liver, adipose tissue and skeletal muscle. Therefore, using all three measures permits, a broader assessment of metabolic status than any of the three alone. HOMA‐IR was calculated as fasting insulin (μU/ml) multiplied by fasting glucose (mg/dl) divided by 405.^24^ The product of fasting plasma glucose × fasting plasma insulin is an index of hepatic IR. Adiponectin acts as an insulin sensitizer by suppressing hepatic glucose production and increasing glucose uptake and fatty acid oxidation in muscles.^25^ Adiponectin is more closely associated with visceral fat than with subcutaneous fat^25^ and is lower in conditions that are associated with IR such as T2D, cardiovascular disease, hypertension and metabolic syndrome.^26^ The ratio of TG to HDL has been associated with IR in white adolescent males and females with obesity^27^ and it reflects the balance between TG intake, TG clearance by peripheral tissues and TG synthesis and export from the liver.^27^ Transfer of triglyceride from VLDL to HDL increases HDL clearance and reduces plasma HDL.^4^

### 2.6 Conventional metabolite analysis

Conventional metabolites, including plasma glucose, HDL, TGs and uric acid were measured with a Beckman Coulter D × C 600 Clinical Analyser using reagents from Beckman (Brea, CA), Coefficients of variation were <5%.

### 2.7 Plasma BCAAs, BCKAs and glutamate/glutamine

BCAAs including valine and leucine/isoleucine; and glutamate/glutamine (5–1000 μmol/l, <15%) were analysed by flow injection electrospray ionization tandem mass spectrometry (MS/MS) using a Waters TQD instrument (Waters, Milford, MA) and quantified by isotope dilution technique as described previously.^1^, ^4^, ^28^, ^29^ Alpha‐keto acids of leucine (α‐keto‐isocaproate, α‐KIC), isoleucine (α‐keto‐β‐methylvalerate, α‐KMV) and valine (α‐keto‐valerate, KIV) were measured by LC–MS/MS. 20 μl of plasma containing isotopically labelled internal standards KIC‐d3, KIV‐5C13 (Cambridge Isotope Laboratories) and KMV‐d8 (Toronto Research Chemicals) were precipitated with 150 μl of 3 M PCA. 200 μl of 25 M *o*‐phenylenediamine (OPD) in 3 M HCl were added to the supernatants and the samples were incubated at 80°C for 20 min. Keto acids were extracted with ethyl acetate as previously described.^5^, ^30^ The extracts were dried under nitrogen, reconstituted in 200 mM ammonium acetate and analysed on a Waters Xevo TQ‐S triple quadrupole mass spectrometer coupled to a Waters Acquity UPLC system. The analytical column (Waters Acquity UPLC BEH C18 Column, 1.7 μm, 2.1 × 50 mm) was used at 30°C, 10 μl of the sample were injected onto the column and eluted at a flow rate of 0.4 ml/min. The gradient consisted of 45% eluent A (5 mM ammonium acetate in water) and 55% eluent B (methanol) for 2 min, followed by a linear gradient to 95% B from 2 to 2.5 min, held at 95% B for 0.7 min, returned to 45% A and then the column was re‐equilibrated at initial conditions for 1 minute. The total run time was 4.7 min. Mass transitions of *m/z* 203 → 161 (KIC), 206 → 161 (KIC‐d3), 189 → 174 (KIV), 194 → 178 (KIV‐5C13), 203 → 174 (KMV) and 211 → 177 (KMV‐d8) were monitored in a positive ion electrospray ionization mode.^31^ Sums of components measured from these panels included the branched‐chain amino acids (BCAAs), comprising the molar sum of Valine + Leucine/Isoleucine; and their cognate branched‐chain keto acids (BCKAs), evaluated as α‐KIC + α‐KMV + α‐KIV. The ratio of BCKA to BCAA was calculated to reflect overall balance between BCAAs and BCKAs.

### 2.8 Statistical analysis

The primary outcome of this study was the correlation between the total BCKA levels and insulin sensitivity. Minimum sample size was calculated to detect correlations of 0.5 or greater between the BCKAs and insulin sensitivity. In linear regression models using 4 explanatory variables with one testing variable, a sample size of 32 provides a correlation of 0.5 with power of 0.828 and *p* < .05. Thus, our sample size of 33 provided adequate statistical power for our primary outcome. In a secondary analysis, we investigated correlations between individual BCKAs including KIV, KIC and KMV and the surrogate measures of insulin sensitivity. We adjusted p‐values for multiple testing to control the familywise error rate using Hochberg Method.^32^ Lastly, given our previous findings showing that BCAAs, glutamate and uric acid constitute a sex‐dependent metabolic ‘signature’ that is positively associated with IR in youth with obesity, we completed a confirmatory analysis to investigate the relationship between BCAAs, glutamate and uric acid and surrogate measures of insulin sensitivity. For primary, secondary and confirmatory analyses, *p* < .05 was considered statistically significant; analyses were performed using SAS version 9.4 (SAS Institute Inc.).

Changes in anthropometric values and changes in BCAAs, BCKAs, glutamate/glutamine, BCKA/BCAA ratio and uric acid during lifestyle intervention were assessed using paired *t*‐tests. Data were stratified by sex to analyse the effects separately for females and males. Unpaired t tests were used to compare anthropometric values and BCAA‐related catabolic byproducts among 17 females and 16 males at baseline and at 6‐month follow‐up.

Multiple linear regression models were used to analyse the associations among changes in BCKAs, glutamate/glutamine and uric acid and changes in surrogate measures of IR and changes in weight. HOMA‐IR, adiponectin and TG/HDL ratio were natural log transformed to approximate normality. In linear regression models, we used one testing variable and 3 confounding variables; these were sex, age and BMI% exceeding the 95th percentile. Thus, all models were adjusted for age, sex and BMI% exceeding the 95th percentile. Models for change in insulin sensitivity were also adjusted for change in BMI% exceeding the 95th percentile. Likewise, the model for change in BMI% exceeding the 95th percentile was also adjusted for change in HOMA‐IR. To investigate if baseline BCKAs, glutamate/glutamine and uric acid predict subsequent changes in insulin sensitivity in response to lifestyle intervention, we used BCKAs, glutamate/glutamine and uric acid at baseline as explanatory variables in linear regression models. In addition, the correlations between the three surrogate measures of IR at baseline and 6‐month‐follow‐up and their changes in response to lifestyle intervention were assessed.

## 3 RESULTS

### 3.1 Response to lifestyle intervention: comparisons of anthropometric values and metabolic characteristics at baseline and follow‐up

As we previously reported, there was large inter‐individual variability in the effect of the lifestyle intervention on physiologic and metabolic parameters measured in this cohort.^4^ The absence of statistical significance for the effect of the intervention on these measures reflects the presence of both responders and non‐responders. In the cohort as a whole, there were no significant changes in BMI‐related metrics, insulin sensitivity measures (as previously noted, Ref. 2), or the levels of BCAAs, BCKAs, BCKA/BCAA ratio, KIV, KIC, KMV, glutamate/glutamine or uric acid (Table 1).

**TABLE 1:** Comparisons of anthropometric values and metabolites, baseline and follow‐up

	Baseline Mean (SE) *n* = 33	Follow‐up Mean (SE) *n* = 33	*p* Value
Anthropometric values			
Age, years	14.20 (0.25)	14.71 (0.25)	**<.0001**
BMI	34.67 (1.17)	34.85 (1.21)	.5108
BMI %	98.31 (0.29)	98.27 (0.24)	.8025
BMI *Z*‐score	2.27 (0.07)	2.25 (0.07)	.3586
BMI% exceeding the 95th percentile	129.75 (4.14)	128.30 (4.24)	.1674
Insulin sensitivity measures			
Adiponectin, μg/ml	15.90 (1.28)	16.31 (1.20)	.6026
HOMA‐IR	4.04 (0.62)	5.20 (1.29)	.1310
TG to HDL ratio	1.85 (0.22)	1.89 (0.26)	.7800
Metabolites			
BCAA, μM	202.58 (5.38)	201.15 (6.33)	.8219
BCKA, μM	71.39 (2.45)	68.53 (2.92)	.3763
BCKA/BCAA	0.35 (0.01)	0.34 (0.01)	.2156
KIV	15.50 (0.45)	14.75 (0.57)	.2743
KIC	33.26 (1.29)	32.50 (1.45)	.6297
KMV	22.63 (0.83)	21.29 (0.98)	.2039
Uric Acid, mg/dl	5.50 (0.22)	5.54 (0.24)	.8096
Glutamate/glutamine μM	35.17 (2.06)	34.08 (1.53)	.4529

Bold *p* value of < 0.0001is statisticaly significant. *p* < 0.05 is considered statsitically significant.

### 3.2 Comparisons of anthropometric values and metabolites by sex

At baseline, males and females were comparable in age, weight and BMI‐related metrics. At baseline and follow‐up, males had higher levels of BCAAs than females (216.10 ± 8.25 μM vs 189.8 ± 5.61 μM at baseline, *p* = .0121 and 215.90 ± 8.95 vs 187.3 ± 7.76 μM at follow‐up; *p* = .0215) and Glutamate/Glutamine (40.24 ± 3.48 μM vs 30.40 ± 1.68 μM at baseline, *p* = .0186 and 38.16 ± 2.50 μM vs 30.24 ± 1.30 μM at follow‐up, *p* = .0101), consistent with our previous findings^1^, ^4^ and with studies in adults with overweight and obesity.^33^ Baseline BCKAs (the total of KIV, KIC and KMV) were also higher among males than females (79.09 ± 3.52 vs 64.13 ± 2.38 at baseline, *p* = .0012). In response to lifestyle intervention, the levels of KMV, the BCKA derived from isoleucine, decreased in males but not in females and the ratio of BCKA/BCAA trended lower in males (Table 2). There was no effect of the intervention on BCAA levels in the cohort as a whole or in males or females analysed separately.

**TABLE 2:** Comparisons of anthropometric values and metabolites across sex, baseline and 6‐month follow‐up

	Baseline male Mean (SE) *n* = 16	Baseline female Mean (SE) *n* = 17	Follow‐up male Mean (SE) *n* = 16	Follow‐up female Mean (SE) *n* = 17	*p* Value^a^	*p* Value^b^	*p* Value^c^	*p* Value^d^
Anthropometric values								
Age, years	14.03 (0.36)	14.36 (0.36)	14.55 (0.36)	14.87 (0.36)	.5192	.5291	<.0001	<.0001
BMI	35.07 (1.80)	34.29 (1.55)	35.00 (1.81)	34.72 (1.69)	.7430	.9095	.8713	.2540
BMI %	98.60 (0.45)	98.04 (0.37)	98.61 (0.31)	97.95 (0.35)	.3424	.1676	.6872	.2775
BMI *Z*‐score	2.38 (0.10)	2.17 (0.08)	2.36 (0.10)	2.14 (0.08)	.1103	.0992	.9621	.4834
BMI% exceeding the 95th percentile	134.80 (6.25)	125.0 (5.39)	132.40 (6.23)	124.50 (5.79)	.2407	.3612	.1563	.6957
Insulin sensitivity measures								
Adiponectin, μg/ml	15.87 (2.03)	15.93 (1.65)	17.45 (2.15)	15.24 (1.19)	.9801	.3774	.1589	.5429
HOMA‐IR	4.72 (1.24)	3.40 (0.31)	5.85 (2.58)	4.58 (0.73)	.3163	.6416	.4438	.0591
TG to HDL ratio	2.22 (0.40)	1.50 (0.19)	2.24 (0.45)	1.57 (0.28)	.1208	.2042	.9270	.7474
Metabolites								
BCAA, μM	216.10 (8.25)	189.8 (5.61)	215.90 (8.95)	187.3 (7.76)	**.0121**	**.0215**	.9771	.7926
BCKA, μM	79.09 (3.52)	64.13 (2.38)	72.97 (2.94)	64.36 (4.82)	**.0012**	.1393	.1092	.9657
BCKA/BCAA	0.37 (0.01)	0.34 (0.01)	0.34 (0.01)	0.34 (0.01)	.0502	.8856	.0565	.9737
KIV	16.73 (0.61)	14.34 (0.53)	15.28 (0.64)	14.24 (0.94)	**.0056**	.3691	.0820	.9276
KIC	37.07 (1.91)	29.68 (1.28)	34.86 (1.43)	30.28 (2.39)	**.0027**	.1127	.2632	.8093
KMV	25.29 (1.18)	20.12 (0.78)	22.83 (1.07)	19.84 (1.57)	**.0008**	.1264	**.0332**	.8746
Uric Acid, mg/dl	5.97 (0.35)	5.07 (0.23)	6.10 (0.35)	5.01 (0.27)	**.0364**	**.0186**	.6091	.6507
Glutamate/glutamine μM	40.24 (3.48)	30.40 (1.68)	38.16 (2.50)	30.24 (1.30)	**.0186**	**.0101**	.4129	.9199

*p* value < 0.05 is considered statistically significant.

^a^ Baseline male vs baseline female.

^b^ Follow‐up male vs follow‐up female.

^c^ Baseline male vs follow‐up male.

^d^ Baseline female vs follow‐up female.

### 3.3 Correlations among surrogate measures of IR

At baseline, there was a significant correlation between HOMA‐IR and TG/HDL (*r* = 0.52, *p* = .0019) in the cohort as a whole. At 6‐month‐follow‐up, there were no significant correlations among the three surrogate measures of IR. Likewise, there were no correlations in the changes in these metrics in response to lifestyle intervention (Table 3).

**TABLE 3:** Correlations between surrogate measures of IR at baseline, 6‐month follow‐up and changes during intervention.

	At baseline			At 6 months			Change from baseline to 6 months		
	HOMA‐IR	Adiponectin	TG to HDL ratio	HOMA‐IR	Adiponectin	TG to HDL ratio	HOMA‐IR	Adiponectin	TG to HDL ratio
At baseline									
HOMA‐IR	1.0000	−0.3369	0.5208	0.7847	−0.3117	0.5004	0.086	0.0538	−0.0073
		.0553	.0019	<.0001	.0774	.0030	.6329	.7662	.9679
Adiponectin	−0.3369	1.0000	−0.3899	−0.1742	0.8309	−0.2944	0.1157	−0.3205	0.1326
	.0553		.0249	.3323	<.0001	.0964	.5213	.0690	.4619
TG to HDL ratio	0.5208	−0.3899	1.000	0.3462	−0.3688	0.7949	−0.0555	0.0485	−0.2769
	.0019	.0249		.0485	.0347	<.0001	.7592	.7885	.1187
At 6 months									
HOMA‐IR	0.7847	−0.1742	0.3462	1.0000	−0.2462	0.2189	0.6854	−0.1170	−0.1849
	<.0001	.3323	.0485		.1672	.2209	<.0001	.5169	.3029
Adiponectin	−0.3117	0.8309	−0.3688	−0.2462	1.0000	−0.2190	−0.0296	0.2607	0.2196
	.0774	<.0001	.0347	.1672		.2207	.8703	.1429	.2195
TG to HDL ratio	0.5004	−0.2944	0.7949	0.2189	−0.2190	1.0000	−0.2360	0.1379	0.3629
	.0030	.0964	<.0001	.2209	.2207		.1861	.4443	.0379
Change from baseline to 6 months									
HOMA‐IR	0.0863	0.1157	−0.0555	0.6854	−0.0296	−0.2360	1.0000	−0.2512	−0.2886
	.6329	.5213	.7592	<.0001	.8703	.1861		.1586	.1033
Adiponectin	0.0538	−0.3205	0.0485	−0.1170	0.2607	0.1379	−0.2512	1.0000	0.1438
	.7662	.0690	.7885	.5169	.1429	.4443	.1586		.4248
TG to HDL ratio	−0.0073	0.1326	−0.2769	−0.1849	0.2196	0.3629	−0.2886	0.1438	1.0000
	.9679	.4619	.1187	.3029	.2195	.0379	.1033	.4248	

Values on the top rows show Pearson Correlation Coefficients and the values on the bottom rows show the *p*‐values.

### 3.4 Correlations among BCAAs, BCKAs and glutamate/glutamine

Glutamate/glutamine levels were positively associated with BCAA levels both at baseline and 6‐month‐follow‐up (*r* = 0.55, *p* = .001; *r* = 0.53, *p* = .0015, respectively). There was no correlation between glutamate/glutamine and BCKA levels. Likewise, there was no correlation between change in glutamate levels and change in BCAA or BCKA levels in response to lifestyle intervention.

### 3.5 Correlations among BCAAs, BCKAs, glutamate/glutamine and BMI% exceeding the 95th percentile

There were relatively strong positive associations between glutamate/glutamine and BMI% exceeding the 95th percentile both at baseline and 6‐month follow‐up (*r* = 0.54, *p* = .0013 and *r* = 0.45, *p* = .0084, respectively). BMI% exceeding the 95th percentile did not correlate significantly with either BCAAs or BCKAs.

### 3.6 Associations between surrogate measures of insulin sensitivity and BCKAs, glutamate/glutamine and uric acid at baseline and 6‐month‐follow‐up

**FIGURE 1:**
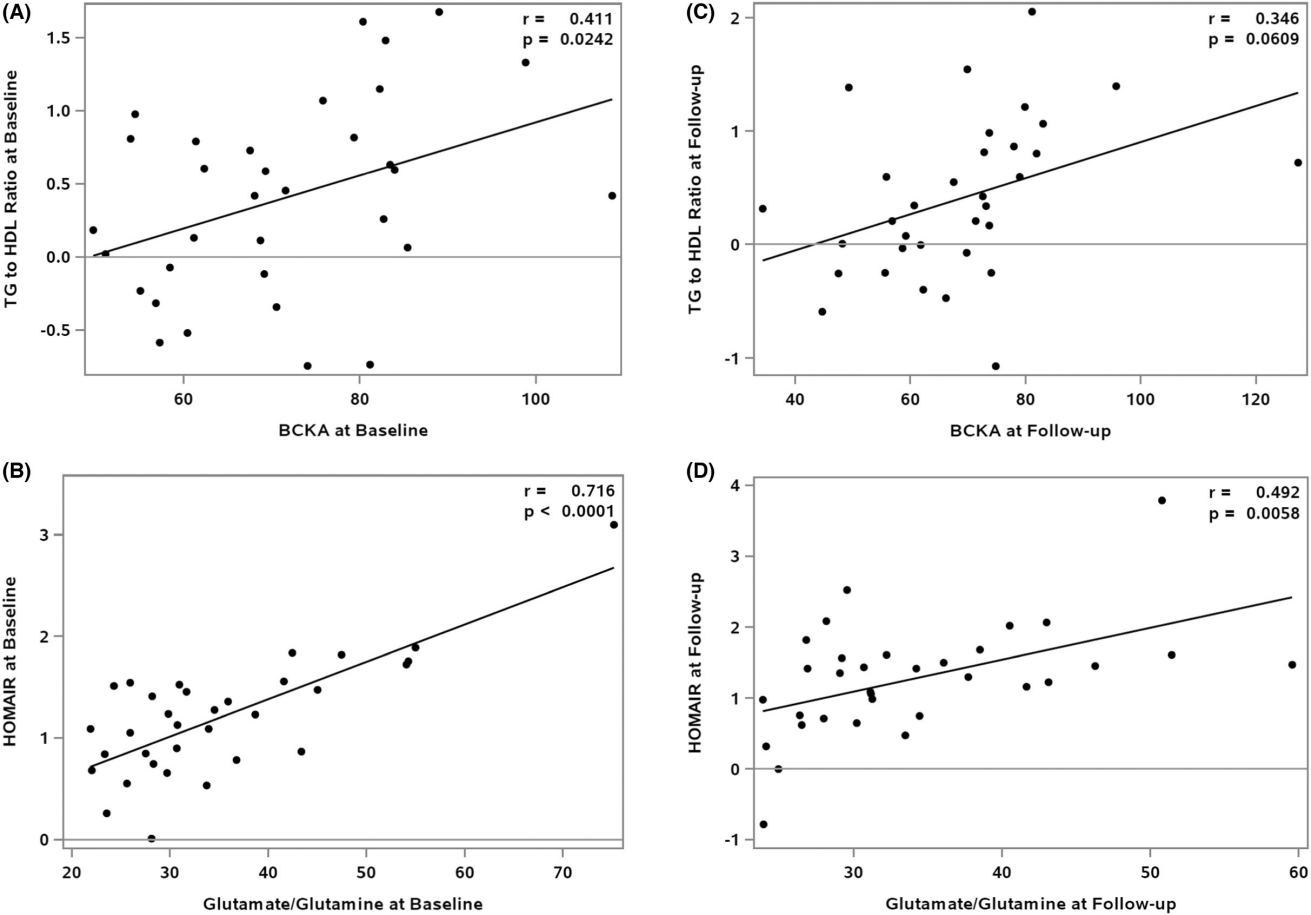
Associations between BCKA and Glutamate, and TG/HDL ratio and HOMA‐IR at baseline and follow‐up, r is the partial Pearson correlation coefficient adjusted for sex, age, BMI% exceeding the 95th percentile. (A) Associations between BCKA and TG/HDL ratio at Baseline, (B) Associations between Glutamate and HOMA‐IR at Baseline, (C) Associations between BCKA and TG/HDL ratio at follow‐up, (D) Associations between Glutamate and HOMA‐IR at Follow‐up

To assess the associations between insulin sensitivity and the various metabolites in the cohort as a whole, we adjusted all models for age, sex and BMI% exceeding the 95th percentile. Baseline total BCKA levels associated positively with baseline TG/HDL ratio (parameter estimate: 0.0194, *p* = .0242) but were not associated with HOMA‐IR or adiponectin (Table 4, Figure 1A). At 6‐month follow‐up, total BCKAs associated positively with HOMA‐IR (parameter estimate: 0.0188, *p* = .0137) and approached significance with the TG/HDL ratio (parameter estimate: 0.0063, *p* = .0609) (Table 4, Figure 1C). KIC and KMV were each positively associated with TG/HDL ratio at baseline (*p* = .0345 and *p* = .0141) and KIV, KIC and KMV correlated positively with HOMA‐IR (*p* = .0287, *p* = .0255 and *p* = .0056, respectively), at 6 months. When models were adjusted for multiple comparisons, KMV had the strongest association with TG/HDL ratio at baseline and HOMA‐IR at 6 months follow‐up (Table 5).

**TABLE 4:** Primary analysis of (A) associations between BCKA and surrogate measures of insulin sensitivity at baseline and 6 months follow‐up; (B) association between change in BCKA and change in adiponectin and (C) baseline BCKA predicting change in adiponectin.

Full Sample (*n* = 33)	Parameter estimate	*t* Value	*p* Value
TG to HDL ratio at baseline	(*R* ^2^ = 0.420, *p* = .038)		
Intercept	−3.3450	−2.83	.0085
Age	0.0285	0.42	.6791
Sex (Female = 1)	0.1669	0.71	.2338
BMI% exceeding the 95th percentile	0.0144	3.44	.0019
BCKA at Baseline	0.0194	2.38	.0242
TG to HDL Ratio at 6 months follow up	(*R* ^2^ = 0.371, *p* = .009)		
Intercept	−2.4136	−2.07	.0477
Age	0.0288	0.40	.6932
Sex (Female = 1)	−0.1086	−0.51	.6150
BMI% exceeding the 95th percentile	0.0125	2.89	.0074
BCKA at 6 months follow‐up	0.0063	1.95	.0609
HOMA‐IR at 6 months follow‐up	(*R* ^2^ = 0.408, *p* = .004)		
Intercept	1.3095	−0.92	.3648
Age	0.0812	−0.88	.3843
Sex (Female = 1)	0.2399	1.72	.0957
BMI% exceeding the 95th percentile	0.0049	3.19	.0035
BCKA at 6 months follow‐up	0.0188	2.63	.0137
Δ Adiponectin	(*R* ^2^ = 0.291, *p* = .041)		
Intercept	−0.5373	−1.27	.2137
Age	0.0503	1.84	.0770
Sex (Female = 1)	−0.1123	−1.39	.1758
BMI% exceeding the 95th percentile	−0.0008	−0.44	.6605
Δ BCKA	−0.0048	−2.21	.0351
Δ Adiponectin Prediction	(*R* ^2^ = 0.343, *p* = .016)		
Intercept	−1.0831	−2.35	.0258
Age	0.0445	1.68	.1048
Sex (Female = 1)	−0.0132	−0.15	.8856
BMI% exceeding the 95th percentile	−0.0010	−0.61	.5440
Δ BCKA	0.0087	2.74	.0106

*Note*: All models were adjusted for sex, age and BMI% exceeding the 95th percentile. Change in metric (Δ) means value at 6 months follow‐up minus value at baseline. The table presents only statistically significant findings. Total BCKA at baseline did not associate with HOMA‐IR or adiponectin at baseline and total BCKA at 6 months follow‐up did not associate with adiponectin at 6 months follow‐up. Change in total BCKA did not associate with change in HOMA‐IR or change in TG/HDL. Total BCKA at baseline did not predict change in HOMA‐IR or TG/HDL.

**TABLE 5:** Secondary analysis of (A) associations between KIV, KIC and KMV and TG/HDL at Baseline and HOMA‐IR at 6 months follow‐up; (B) associations between changes in KIV, KIC and KMV and changes in adiponectin and (C) baseline KIV, KIC and KMV predicting change in adiponectin.

Full Sample (*n* = 33)	Parameter estimate	*t* Value	*p* value	Adj *p* value
TG to HDL ratio at baseline				
KIV at baseline (*R* ^2^ = 0.400, *p* = .013)	0.0714	1.59	.1229	.1229
KIC at baseline (*R* ^2^ = 0.400, *p* = .005)	0.0341	2.22	.0345	.0690
KMV at baseline (*R* ^2^ = 0.434, *p* = .003)	0.0634	2.62	.0141	.0423
HOMA‐IR at 6 months follow‐up				
KIV at 6 months follow‐up (*R* ^2^ = 0.379, *p* = .008)	0.0864	2.31	.0287	.0287
KIC at 6 months follow‐up (*R* ^2^ = 0.384, *p* = .007)	0.0348	2.36	.0255	.0287
KMV at 6 months follow‐up (*R* ^2^ = 0.441, *p* = .002)	0.0623	3.00	.0056	.0168
Δ Adiponectin				
Δ KIV (*R* ^2^ = 0.313, *p* = .028)	−0.0246	−2.44	.0215	.0574
Δ KIC (*R* ^2^ = 0.300, *p* = .035)	−0.0101	−2.31	.0287	.0574
Δ KMV (*R* ^2^ = 0.246, *p* = .086)	−0.0120	−1.71	.0986	.0986
Δ Adiponectin prediction				
KIV at baseline (*R* ^2^ = 0.347, *p* = .015)	0.0461	2.78	.0097	.0194
KIC at baseline (*R* ^2^ = 0.347, *p* = .015)	0.0164	2.78	.0096	.0194
KMV at baseline (R^2^ = 0.276, *p* = .053)	0.0207	2.06	.0490	.0490

*Note*: All models were adjusted for sex, age and BMI% exceeding the 95th percentile. Parameter estimates are presented only for the hypothesis testing variable. *p*‐Values were adjusted for multiple testing to control the familywise error rate using Hochberg Method. Change in metric (Δ) means values at 6 months follow‐up minus values at baseline.

Glutamate/glutamine was positively associated with HOMA‐IR both at baseline and 6‐months follow‐up (parameter estimate: 0.0365, *p* < .0001 and parameter estimate 0.0474, *p* = .0058) (Table 6, Figure 1B, D) but not with TG/HDL ratio or adiponectin levels. Since BCAA levels were positively correlated with glutamate/glutamine levels, we also adjusted the models for BCAA levels in addition to age, sex and BMI% exceeding the 95th percentile. Even after adjustment for BCAA levels, the levels of glutamate/glutamine associated positively with HOMA‐IR at baseline (*r* ^2^ = 0.68, *p* < .0001) and approached significance at 6‐month follow‐up (*r* ^2^ = 0.56, *p* = .0773). Uric acid did not correlate with surrogate measures of insulin sensitivity.

**TABLE 6:** Confirmatory Analysis of (A) associations between GLX, uric Acid, BCAA and insulin sensitivity at baseline and 6 months follow‐up; (B) associations between changes in GLX, Uric Acid, BCAA and changes in surrogate measures of insulin sensitivity and (C) baseline GLX, Uric Acid, BCAA predicting change in adiponectin and change in BMI% exceeding the 95th percentile.

Full sample (*n* = 33)	Parameter estimate	*t* Value	*p* Value
HOMA‐IR at baseline			
GLX at baseline (*R* ^2^ = 0.678, *p* = .001)	0.0365	5.43	<.0001
TG to HDL Ratio at 6 months follow‐up			
BCAA at 6 months follow‐up (*R* ^2^ = 0.379, *p* = .008)	0.0063	2.06	.0493
HOMA‐IR at 6 months follow up			
BCAA at 6 months follow‐up (*R* ^2^ = 0.503, *p* = .001)	0.0118	3.69	.0009
GLX at 6 months follow‐up (*R* ^2^ = 0.440, *p* = .002)	0.0474	2.99	.0058
Δ Adiponectin prediction			
BCAA at baseline (*R* ^2^ = 0.290, *p* = .042)	0.0031	2.20	.0364
Δ BMI% exceeding the 95th percentile prediction			
Uric acid at baseline (*R* ^2^ = 0.341, *p* = .017)	−3.4446	−3.64	.0011

*Note*: All models were adjusted for sex, age and BMI% exceeding the 95th percentile. Change in metric (Δ) means values at 6 months follow‐up minus values at baseline.

### 3.7 Associations between changes in weight and insulin sensitivity and changes in BCKAs, glutamate/glutamine and uric acid

**FIGURE 2:**
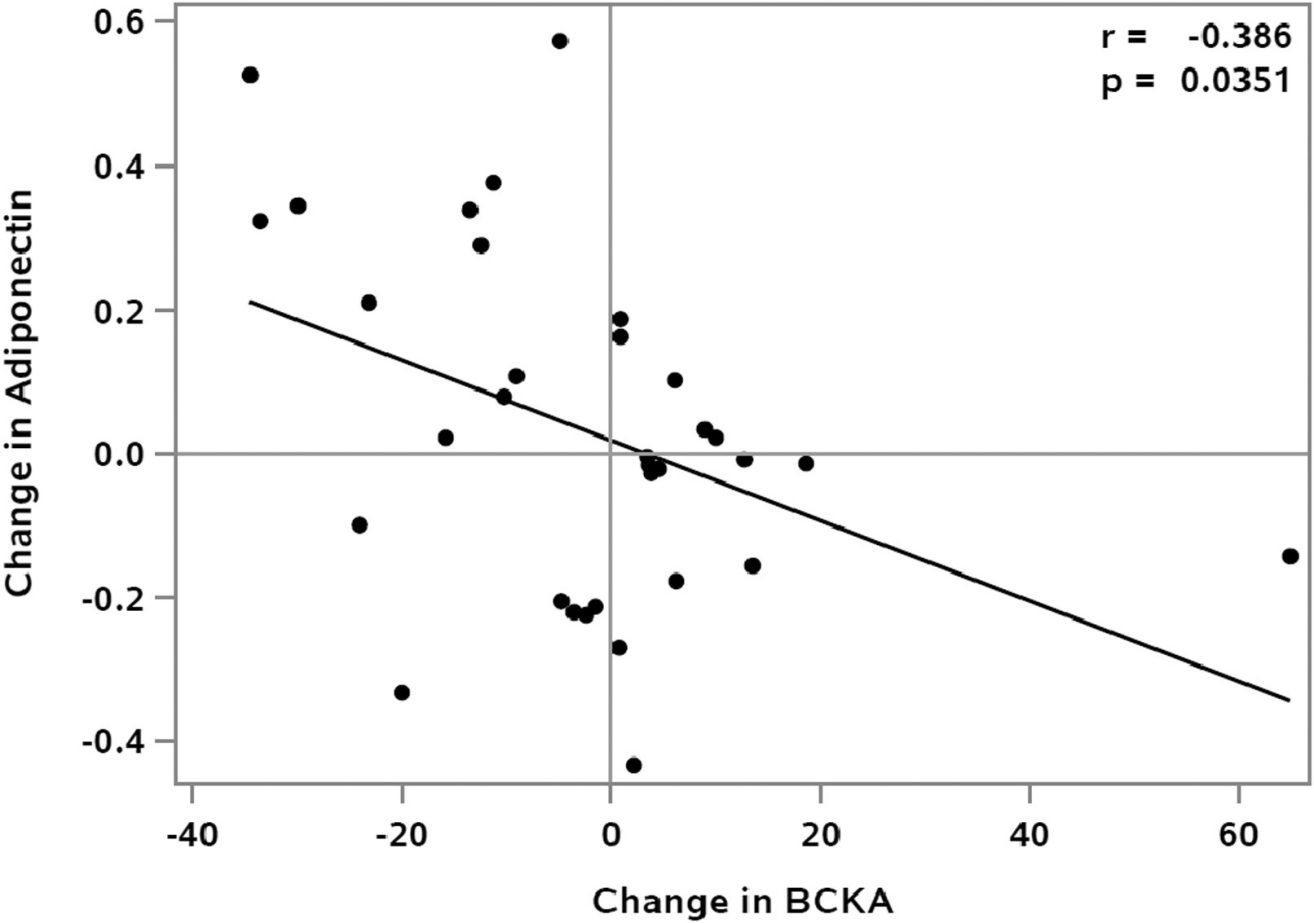
Associations between Change in BCKA and Change in Adiponectin, r is the partial Pearson correlation coefficient adjusted for sex, age, BMI% exceeding the 95th percentile.

In a previous investigation,^4^ we found that reductions in weight in response to lifestyle counselling were associated with reductions in circulating BCAA and catabolic byproducts and increases in adiponectin. Here, a reduction in total BCKAs during lifestyle intervention was associated with an increase in adiponectin (*p* = .035) (Table 4, Figure 2). Likewise, reductions in KIV and KIC were associated with increases in adiponectin (*p* = .022 and .029, respectively) (Table 5). When models were adjusted for multiple comparisons, p values approached significance (Table 5).

To determine if associations between BCKAs and insulin sensitivity were mediated by changes in weight, we adjusted models of change in insulin sensitivity for change in BMI% exceeding the 95th percentile. When the model for change in adiponectin was adjusted for change in BMI% exceeding the 95th percentile, the change in total BCKAs and changes in KIV and KIC remained significant (*p* = .0449, *p* = .0264 and *p* = .0369, respectively). These findings suggest that change in adiponectin is mediated by, or associated with changes in BCKAs that are independent, at least in part, of change in BMI% exceeding the 95th percentile. There were no significant correlations between changes in total or individual BCKAs and changes in other surrogate measures of insulin sensitivity (HOMA‐IR and TG/HDL ratio) or weight.

Changes in uric acid or glutamate/glutamine were not associated with changes in surrogate measures of insulin sensitivity or weight.

### 3.8 Prediction models

#### 3.8.1 Did baseline BCKAs, glutamate/glutamine and uric acid predict subsequent changes in weight or markers of insulin sensitivity?

**FIGURE 3:**
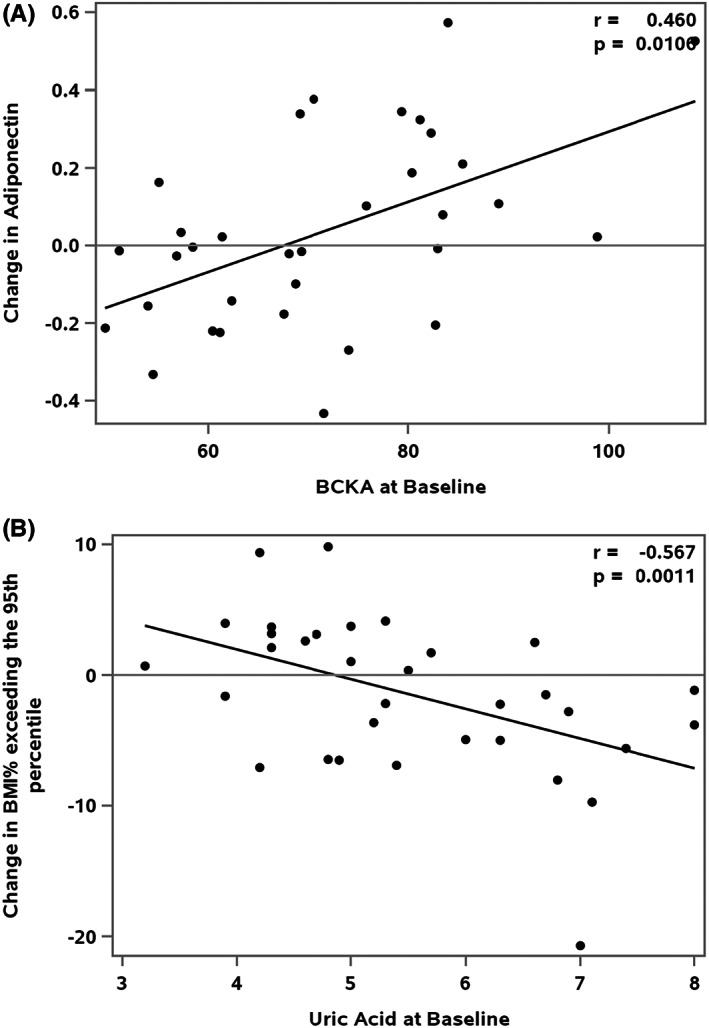
Prediction of Adiponectin and BMI% exceeding the 95th percentile with BCKA and Uric Acid, r is the partial Pearson correlation coefficient adjusted for Sex, Age, BMI% exceeding the 95th percentile at baseline. (A) Prediction of Adiponectin with BCKA, (B) Prediction of BMI% exceeding the 95th percentile with uric acid.

Tables 4, 5, 6 provides the selected prediction models for subsequent changes in weight and insulin sensitivity. Participants with higher total BCKAs at baseline had larger increases in adiponectin levels at follow‐up (parameter estimate: 0.0087, *p* = .0106, Figure 3A). Likewise, higher KIV, KIC and KMV levels at baseline predicted a greater increase in adiponectin at 6‐month follow‐up (*p* = .0097, *p* = .0096 and *p* = .0490, respectively), even after adjustment for multiple comparisons (*p* = .0194, *p* = .0194 and *p* = .0490, respectively). Baseline glutamate/glutamine levels did not predict subsequent changes in weight or insulin sensitivity. Interestingly, those with higher baseline uric acid levels had greater weight reduction (Table 6, parameter estimate: −3.4446, *p* = .0011, Figure 3B). However, baseline uric acid levels did not predict changes in insulin sensitivity.

## 4 DISCUSSION

Studies in children and adolescents suggest that BCAAs are associated with IR and T2D and predict future risk of IR, metabolic syndrome and hypertriglyceridemia.^1^, ^34^, ^35^, ^36^, ^37^, ^38^, ^39^ Studies in adults suggest that elevated plasma BCKAs are also associated with IR and predict more reliably than BCAAs the development of T2D.^6^, ^7^ BCKAs are also associated with other obesity co‐morbidities such as steatosis and NASH.^40^ In this study, we measured levels of BCAA, BCKA, glutamate/glutamine and uric acid prior to and following a 6‐month course of lifestyle intervention and assessed their relationships to changes in weight and metrics of insulin sensitivity. We then determined if levels of BCKAs, glutamate/glutamine and uric acid at baseline predicted changes in weight or insulin sensitivity. Finally, we investigated if BCKAs are regulated in a sex‐dependent manner such as the BCAAs, glutamate/glutamine and uric acid.

Our findings include four novel observations*. First*, the BCKAs, particularly KMV, correlated positively with TG/HDL (at baseline) and HOMA‐IR (at 6‐month‐follow‐up), while glutamate/glutamine associated strongly with HOMA‐IR both at baseline and following lifestyle intervention. Thus, byproducts of BCAA catabolism, particularly Glutamate/glutamine, were associated with insulin sensitivity both at baseline and follow‐up (Table 6). *Second*, a reduction in BCKAs during lifestyle intervention was associated with an increase in adiponectin, a metric of improved insulin sensitivity (Table 4). *Third*, high BCKA levels at baseline predicted higher adiponectin levels at follow‐up (Table 4), and, those with higher uric acid levels at baseline had greater weight reduction (reduction in BMI% exceeding the 95th percentile) in response to lifestyle intervention (Table 6). *Fourth*, sex differences in the metabolites at baseline persisted during intervention: baseline BCKAs (KIV, KIC and KMV), glutamate/glutamine and uric acid were higher among males than females. In response to lifestyle intervention, the levels of KMV, a metabolic by product of isoleucine, decreased in males but not in females and the molar ratio of BCKA/BCAA trended lower in males (Table 2). Notably, in males the impact of the intervention on BCKAs occurred absent any effect on BCAA levels.

We do not yet know why those with highest baseline BCKAs had the greatest increases in adiponectin in response to lifestyle intervention. However, our observation is consistent with previous studies in adults and children with obesity showing that baseline levels of a BCAA‐related PCA‐factor predicts improvements in insulin sensitivity in response to behavioural weight loss interventions.^4^, ^41^, ^42^ We speculate that lifestyle counselling may have had its greatest impact in the most insulin resistant participants with the lowest BCKDH activity and highest levels of BCKAs. It is possible that high BCKA levels at baseline may reflect low adiponectin levels, given that adiponectin is proposed to activate hepatic BCKDH.^43^

Interestingly, while both BCKAs and BCAAs are elevated in adults with obesity,^7^, ^41^, ^42^, ^44^ a recent analysis found that BCKAs were lower in adolescents with obesity than healthy weight controls.^45^ The participants in that cohort were younger (12.6 year vs 14.2 year) and sexually less mature than our participants. This observation suggests that there may be developmental changes in the regulation of BCAT (which generates BCKA from BCAA) and BCKDH (which catalyses the oxidative decarboxylation of BCKA) during the transition from early adolescent to mature adolescent/adult adiposity. The role of sex steroids in those developmental changes requires further evaluation.

That changes in metabolites derived from BCAA catabolism correlated with changes in insulin sensitivity in our study is not surprising given genetic evidence of a causal effect of IR on BCAA levels.^46^, ^47^ Genetic and acquired variations in BCAA catabolism in adults are also associated with higher risk for T2D but not IR which suggests that impaired BCAA catabolism may exacerbate metabolic dysfunction in the setting of IR.^48^ In the TwinsUK study, SNP rs1440581 had the strongest associations with all BCAAs, all BCKAs and the C3‐acylcarnitine propionylcarnitine and was associated with higher T2D risk.^7^ This SNP is upstream of PPM1K mitochondrial phosphatase, which dephosphorylates and activates BCKDH.^7^ Accordingly, studies in genetically obese rats (fa/fa) and (ob/ob) mice demonstrate that activation of BCKDH through pharmacological inhibition of the BCKDH kinase or overexpression of the PPM1K phosphatase reduced the abundance of BCAAs and BCKAs and markedly attenuated IR.^5^ Moreover, exposure of the isolated perfused heart to levels of BCKA found in obese rodents is sufficient to cause phosphoproteome remodelling and activate protein synthesis.^49^ Also, exposure to high levels of BCKAs has also been shown to suppress insulin signalling via activation of mammalian target of rapamycin complex 1,^6^, ^50^ although unlike the perfused heart study, these latter two studies used supraphysiologic doses of BCKAs that may not reflect in vivo concentrations. Thus, elevated circulating BCKAs may be both biomarkers and causal agents of insulin resistance and other cardiometabolic disease phenotypes.^51^

Of the three BCKAs measured in our study, KMV was more strongly associated with TG/HDL ratio at baseline and HOMA‐IR at 6 months follow‐up than either KIV or KIC (Table 5). It is unclear why the BCKA derived from isoleucine is most strongly associated with IR. However, these data are consistent with a large population‐based cohort in adults in which the KMV was also found to be the strongest predictive biomarker for impaired fasting glucose.^7^ The association was replicated in an independent population. These results suggest a unique role for impaired catabolism of isoleucine in IFG and T2D.

In response to lifestyle intervention, the levels of KMV decreased in males but not females and the ratio of BCKA/BCAA trended lower in males (Table 2). Thus, in males, there was a greater impact of the intervention on BCKAs than on BCAAs. This suggests sex‐dependent differences in regulation of BCAT and BCKDH. Sex differences in circulating BCKAs, glutamate/glutamine and uric acid levels in teens with obesity are currently unexplained but could in theory reflect sex differences in BCAA production by the microbiome, and/or differences in rates of protein synthesis or proteolysis or BCAA catabolism.^5^, ^52^, ^53^, ^54^ The roles of sex steroids in BCAA production or catabolism are poorly understood but could in theory be mediated by effects on fat distribution and liver fat deposition: testosterone promotes visceral fat deposition and hepatic fat accumulation while oestrogen increases subcutaneous fat storage and limits hepatic steatosis.^55^, ^56^, ^57^ It is unclear whether the sex differences in BCAA‐related metabolites that we observed contribute to differences in the risks of T2D in adolescent males and females and/or differences in their responses to pharmacological agents.

Glutamate is produced via multiple transamination reactions including the BCAT‐dependent step of BCAA catabolism.^42^ An increase in plasma glutamate may enhance insulin secretion since glutamate can serve as an insulin secretagogue via its conversion to α‐ketoglutarate by glutamate dehydrogenase^58^, ^59^; it is also a substrate for hepatic gluconeogenesis.^60^ Stefan et al. recently found that elevated plasma glutamate levels are associated with higher liver fat content, lower insulin sensitivity and increased carotid intima media thickness independent of total body fat mass and visceral fat mass.^14^ In their study, glutamate and glutamine levels were measured separately; metabolic dysfunction associated most strongly with glutamate. Nevertheless, plasma levels of glutamine also correlated with liver fat content and negatively with insulin sensitivity metrices derived from clamp studies, and both glutamate and glutamine levels correlated with BCAAs. Likewise, we found that glutamate/glutamine levels correlated positively with BCAA levels and with BMI% exceeding the 95th percentile. Glutamate/glutamine associated with HOMA‐IR even after adjustment for BCAAs as well as age, sex and BMI% exceeding the 95th percentile. These findings suggest that glutamate/glutamine may have effects on insulin sensitivity that are independent, at least in part, from changes in the BCAA.

We previously showed that glutamate/glutamine and uric acid are part of the sex‐dependent BCAA‐related metabolic ‘signature’ associated with IR in adolescents with obesity.^1^, ^4^ This is interesting given that uric acid is a potential risk factor for developing diabetes, hypertension, stroke and cardiovascular diseases not only in adults but also in adolescents.^15^, ^16^, ^17^ An observational study from the treatment options for type 2 diabetes in adolescents and youth study found elevated serum uric acid levels to be associated with greater risk for hypertension and diabetic kidney diseases in adolescents with type 2 diabetes.^15^ Hyperuricemia is thought to result from decreased insulin‐dependent renal tubular uric acid excretion and/or increased fructose‐dependent uric acid production.^18^, ^19^ However, studies in subjects with gout suggest that increases in glutamate resulting from decreased activity of glutamate dehydrogenase may play a role in the pathogenesis of hyperuricemia.^20^ Thus, the elevated glutamate/glutamine and uric acid we observe in IR in adolescents with obesity might be biologically related. Nevertheless, we found no association between uric acid and measures of insulin sensitivity. However, higher baseline uric acid levels predicted a greater weight reduction in the subjects in our cohort. In this regard, one prospective observational study found a correlation between weight loss and reduction in uric acid.^61^ Thus, baseline uric acid levels might serve as a predictor of weight loss and response to lifestyle intervention.

As in our previous studies,^1^, ^4^ we used HOMA‐IR, adiponectin and the TG/HDL ratio as surrogate measures of IR. These surrogate measures of IR reflect distinct, but overlapping, components of insulin sensitivity regulated at the level of the liver, adipose tissue and skeletal muscle. Given the differential regulation of BCAA catabolism across various tissues in states associated with obesity,^52^, ^53^, ^54^ it may not be surprising that correlations between BCKAs and glutamate/glutamine and HOMA‐IR, adiponectin and TG/HDL varied in response to lifestyle intervention. Consistent with our findings, the circulating metabolic profiles of adults with hepatic and skeletal muscle IR are distinct.^62^

Our study has limitations. The sample size was small but provided adequate statistical power for our primary outcome. Female participants were not studied at standard phases of the menstrual cycle. We used surrogate measures of insulin sensitivity; additional methods, including insulin and glucose clamps and iv and oral glucose tolerance tests, might have provided useful information regarding insulin secretion and tissue‐specific IR.^62^, ^63^ As discussed in detail above, glutamate/glutamine levels were measured together in our mass spectrometry assays.^1^, ^3^ Finally, multiple statistical comparisons and associations may lead to spurious conclusions. To address this, we used Hochberg method to adjust for multiple comparisons for our secondary outcomes. Moreover, most of the significant findings in our confirmatory analyses had p values <0.01. Nevertheless, the exploratory nature of our study requires that our findings be interpreted with caution.

In summary, our findings provide insights into the effects of lifestyle intervention on branched‐chain amino acid‐related metabolites and their associations with insulin sensitivity in adolescents with obesity. Our results suggest that BCKAs (particularly KMV) and glutamate/glutamine might serve as the biomarkers of IR in adolescents with obesity, while uric acid might serve to predict weight loss in response to lifestyle intervention. It remains unclear if increases in BCAA and/or BCKAs are a consequence or cause (or both) of IR in adolescents; indeed, high levels of BCAA might serve an adaptive function, increasing insulin secretion as a response to obesity‐associated IR.^64^ Finally, as discussed here and in our previous manuscripts,^1^, ^4^ BCAAs and their metabolites are regulated differentially among adolescent males and females. Differential regulation of BCAA catabolism in adolescent males and females implicates critical roles for sex steroids in metabolic homeostasis.

## AUTHOR CONTRIBUTIONS

**Pinar Gumus Balikcioglu:** Conceptualization (lead); data curation (lead); formal analysis (lead); funding acquisition (lead); investigation (lead); methodology (lead); project administration (lead); resources (lead); supervision (lead); writing – original draft (lead); writing – review and editing (lead). **Catherine Jachthuber Trub:** Data curation (supporting); investigation (supporting); methodology (supporting); writing – review and editing (supporting). **Metin Balikcioglu:** Data curation (supporting); formal analysis (supporting); project administration (supporting); writing – review and editing (supporting). **Olga Ilkayeva:** Data curation (supporting); formal analysis (supporting); methodology (supporting); writing – review and editing (supporting). **Phillip White:** Data curation (supporting); methodology (supporting); writing – review and editing (supporting). **Michael Muehlbauer:** Data curation (supporting); formal analysis (supporting); methodology (supporting); project administration (supporting); software (lead); validation (lead); writing – review and editing (supporting). **James Bain:** Data curation (supporting); methodology (supporting); resources (supporting); writing – review and editing (supporting). **Sarah Armstrong:** Data curation (supporting); methodology (supporting); project administration (supporting); resources (supporting); writing – review and editing (supporting). **Michael Freemark:** Conceptualization (supporting); data curation (supporting); investigation (supporting); methodology (supporting); project administration (supporting); writing – original draft (supporting); writing – review and editing (supporting).

## FUNDING INFORMATION

P.G.B. was supported by National Institute of Diabetes and Digestive and Kidney Diseases of the National Institutes of Health under the award number DK117067, Children's Miracle Network Hospitals partnerships and programs benefiting Duke Children's, Derfner Foundation Research Grant, and Duke University Pediatric Departmental Support, Duke Strong Start Award Program. PJW was supported by A Pathways to Stop Diabetes Award #1‐16‐INI‐17 from the American Diabetes Association. OI and JB and metabolomics assays that they performed were supported by National Institute of Diabetes and Digestive and Kidney Diseases of the National Institutes of Health under award number P30DK124723. JB also received salary support from J from NIH 5R01DK117491, 1U24DK129557 and 2P30AG027816. The content is solely the responsibility of the authors and does not necessarily represent the official views of the National Institutes of Health.

## CONFLICT OF INTEREST

CJT, MB, JB, MM, OI, SA and PGB have no conflicts of interest to declare. MF is a co‐investigator on a grant from the American Heart Association that deals with the pathogenesis and treatment of childhood obesity. MF is also the local PI on a rhythm‐sponsored study of identification and treatment of children and adults with monogenic obesity and was previously a member of a Data Safety Monitoring Board for a separate Rhythm‐sponsored study of treatment of patients with syndromic obesity. PJW reports a pending patent for metabolic biomarkers of NAFLD/NASH and related disease phenotypes and a pending patent for compositions and methods for treating NAFLD/NASH and related disease phenotypes.

## ETHICAL APPROVAL

The Institutional Review Board at Duke University approved the research protocol.

## Data Availability

The data that support the findings of this study are available from the corresponding author upon reasonable request.
